# Relationships among lightness illusions uncovered by analyses of individual differences

**DOI:** 10.1167/jov.25.12.14

**Published:** 2025-10-07

**Authors:** Yuki Kobayashi, Arthur G. Shapiro

**Affiliations:** 1 Department of Computer Science, American University, Washington DC, USA; 2 Research Organization of Open Innovation and Collaboration, Ritsumeikan University, Osaka, Japan; 3 Department of Psychology, American University, Washington DC, USA; 4 Present address: National Intitute of Information and Communications Technology, Osaka, Japan

**Keywords:** lightness/brightness perception, illusions, computational models, factor analysis, individual differences

## Abstract

Computational models that explain lightness/brightness illusions have been proposed. These models have been assessed using a simplistic criterion: the number of illusions each model can correctly predict from the test set. This simple method of evaluation assumes that each illusion is independent; however, because the independence and similarity among lightness illusions have not been well established, potential interdependencies among the illusions in the test set could distort the evaluation of models. Moreover, evaluating models with a single value obscures where the model's strengths and weaknesses lie. We collected the magnitudes of various lightness illusions through two online experiments and applied exploratory factor analyses. Both experiments identified some underlying factors in these illusions, suggesting that they can be classified into a few distinct groups. Experiment 1 identified three common factors; assimilation, contrast, and White's effect. Experiment 2, with a different illusion set, identified two factors—assimilation and contrast. We then examined three well-known models that are based on early visual processes, using the outcomes of the experiments. The examination of these models revealed biases in the models toward specific factors or sets of illusions, which suggested their limitations. This study clarified that correlations of illusion magnitudes provide valuable insights into both illusions and models and highlighted the need to assess models based on their ability to account for underlying factors rather than individual illusions.

## Introduction

Lightness illusions present two patches that appear to be different achromatic shades even though those same patches appear identical when placed in another context. The illusions are objects of scientific study because they demonstrate that our perception of an object is not determined solely by the light intensity received by the eyes from that object but also depends upon the context in which the object is presented. Researchers have reported numerous lightness illusions (Adelson, 1993; Anderson, 1997; Gilchrist, 1977; Knill & Kersten, 1991) that can be roughly categorized into two groups: effects of context produce either a contrastive effect, in which the lightness of the object stands out more from its immediate surroundings (i.e., a gray field appears lighter against a dark field, and darker against a bright field) (Rudd, 2010; Wallach, 1948; Yund & Armington, 1975) or an assimilative effect, where the object blends more with the surrounding field (i.e., a dark object appears darker against a dark field, and a light object appears lighter against a light surround) (Agostini & Galmonte, 2002a; Bressan, 2001; Gilchrist & Annan, 2002). There are some lightness illusions that seem not to fit into these two categories (Agostini & Galmonte, 2002b; Kobayashi & Morikawa, 2019). It is not easy to determine how context affects lightness since an object's appearance can be affected by both adjacent and distant regions (Adelson, 2000).

The evaluation of models that predict human lightness perception typically start with a sample set of illusions, and then rank the models based on the proportion of illusions they correctly identify the direction of change (i.e., which of the two targets appears lighter/darker) (Bressan, 2006; Corney & Lotto, 2007; Kim, Gold, & Murray, 2018; Kobayashi & Kitaoka, 2022; Murray, 2020; Nedimović, Zdravković, & Domijan, 2022; Robinson, Hammon, & de Sa, 2007; Zeman, Brooks, & Ghebreab, 2015). There are two (somewhat obvious) problems of using the approach as a benchmark of lightness models. The first problem has to do with the independence of illusions in the sample set. Although there are many instances of visual illusion, it is likely that many of these are driven by the same underlying mechanism (Adams, 2007), and this potential commonality might distort the model evaluation by naïve counting. For example, even if a model can predict five illusions, if four of those are driven by the same mechanism, then the model essentially accounts for only two mechanisms. If another model can explain three independent illusions, it should be considered superior to the first one.

The second problem has to do with evaluating models with a single value (the number of correct predictions) instead of multiple metrics. This may obscure where the model's expertise and weakness lie. If we can identify classes of “similar” illusions, we can then determine which classes a mode excels or fails short in predicting. The validity of the naïve counting needs to be examined with more comprehensive understanding regarding the similarities and differences among lightness illusions.

The present study aimed to clarify the relationships among known lightness illusions using correlations of illusion magnitudes. There have been discussions about the theoretical classification of lightness illusions (Agostini, Murgia, Sors, Prpic, & Galmonte, 2020; Gilchrist, 2006), but no attempts have been made to classify them with empirical data. Similar illusions that share an underlying process should exhibit consistent tendencies in individual differences; that is, an individual who experiences a strong effect in one illusion is likely to perceive a correspondingly strong effect in another similar illusion. Correlations of responses to visual tasks have been used for examining the similarity and the difference among several visual processes (Adams, 2007; Emery, Volbrecht, Peterzell, & Webster, 2023; Kobayashi, Zavagno, & Morikawa, 2021a; Llamas-Cornejo, Peterzell, & Serrano-Pedraza, 2024; Morikawa, 2003; Shaqiri et al., 2019) and for identifying common underlying mechanisms of them (Coren, Girgus, Erlichman, & Hakstian, 1976; Kaneko, Murakami, Kuriki, & Peterzell, 2018; Mollon, Bosten, Peterzell, & Webster, 2017; Peterzell, 2016; Peterzell, Serrano-Pedraza, Widdall, & Read, 2017; Peterzell, Werner, & Kaplan, 1995; Takahashi, Ujiie, & Dai, 2023). Measuring correlations of illusion magnitudes and classifying lightness illusions will enable us (1) to examine redundancy in an illusion set and (2) to give more detailed evaluations of models with information about which class of “similar” illusions are explained well. Moreover, organizing the relationships among illusions can 3) offer insights into establishing a standard set of illusions commonly used for model evaluations (Murray, 2021; Schmittwilken, Maertens, & Vincent, 2023).

In this article, we measured magnitudes of a set of lightness illusions and ran exploratory factor analyses (EFA) on the data. EFA assumes that responses to each item (e.g., task or question) vary across individuals and that responses to items driven by a common underlying mechanism should be positively correlated across participants. Therefore, from illusion magnitude correlations, EFA can reveal a small number of common factors affecting a larger number of illusions and to quantify both the influence of these factors on individual illusions (i.e., factor loading) and the extent of their independence from these factors (i.e., uniqueness). Because there is some flexibility in how factor loadings and the number of factors are defined, it is possible to qualitatively categorize illusions by strategically defining the factors (i.e., rotation) such that each illusion has a high loading on only one factor. In psychological studies, EFA is commonly used to classify questionnaire items based on response correlations. Following recent attempts (Cretenoud et al., 2019; Grzeczkowski, Cretenoud, Herzog, & Mast, 2017; Kaneko et al., 2018; Peterzell, 2016; Peterzell et al., 2017), we applied this method to psychophysical experiments to classify illusions.

Others have investigated correlations between different mostly geometrical illusions that involve size, length, angle, or position perception and the results have shown that inter-correlations (correlations between distinct illusions) are weak while intra-correlations (correlations between an illusion and its slightly modified version) are robust (Cretenoud, Francis, & Herzog, 2020; Cretenoud, Grzeczkowski, Bertamini, & Herzog, 2020; Cretenoud et al., 2019; Grzeczkowski, Clarke, Francis, Mast, & Herzog, 2017). These studies used illusions that involve various geometrical aspects but our focus was exclusively on lightness and this increases the likelihood of identifying common factors among illusions that were thought to be “different.” We used open data provided by a previous study (Nedimović et al., 2022) and conducted two online experiments with larger sample sizes. We found that known illusion sets can be classified into a few groups.

## Analysis of open data

Nedimović et al. (2022) measured the magnitudes of lightness illusions using an online experiment with 85 participants. The authors employed eleven lightness illusions and two Mondrian images, and they compared the data of the human observers with the outputs from several computational models. This open dataset was useful in confirming that the correlations of illusion magnitudes are sufficiently informative to reveal their relationships.

We focused our analysis on the eleven illusory images (Figures 2A–K in Nedimović et al., 2022), motivated by our interest in the correlations between illusion magnitudes. These images include four instances of simultaneous lightness contrast (SLC), three variants of White's effect (Clifford & Spehar, 2003; White, 1979; White, 1981), and four illusions where the targets appear to have a luminance closer to that of their immediate surroundings (Bindman & Chubb, 2004; DeValois & DeValois, 1990; Economou, Zdravković, & Gilchrist, 2015; Gilchrist & Annan, 2002). The authors posited that two of the four illusions (Reversed Contrast and Dungeon Illusion) are driven by perceptual grouping, similar to White's effect, and the remaining two (Checkerboard Illusion and Bullseye Illusion) predominantly rely on assimilation.^1^ Therefore they suggested that the 11 illusions could be composed of three primary effects: (1) the contrast effect, where the appearance of a target is distorted away from its immediate surroundings; (2) the reverse contrast effect, where the appearance of a target is distorted away from its perceptually grouped and remotely located surroundings; and (3) the assimilation effect, where the appearance of a target is distorted toward its immediate surroundings. This classification is grounded in existing literature and theoretical. Although the authors primarily analyzed the averages of illusion magnitudes, they did not explore the correlations and classifications using a quantitative approach.

### Methods and results

The dataset from Nedimović et al. (2022) includes the perceived lightness (0-1 scale) of two target areas in each of the eleven images, which was collected from 85 observers through the adjustment task conducted online. It was composed of 1870 data points (11 images × 2 targets × 85 participants). Differences in the perceived lightness of the two targets define the magnitudes of the illusions. The authors reported that all eleven illusions exhibited significant effects in the expected directions.

**Figure 1.**
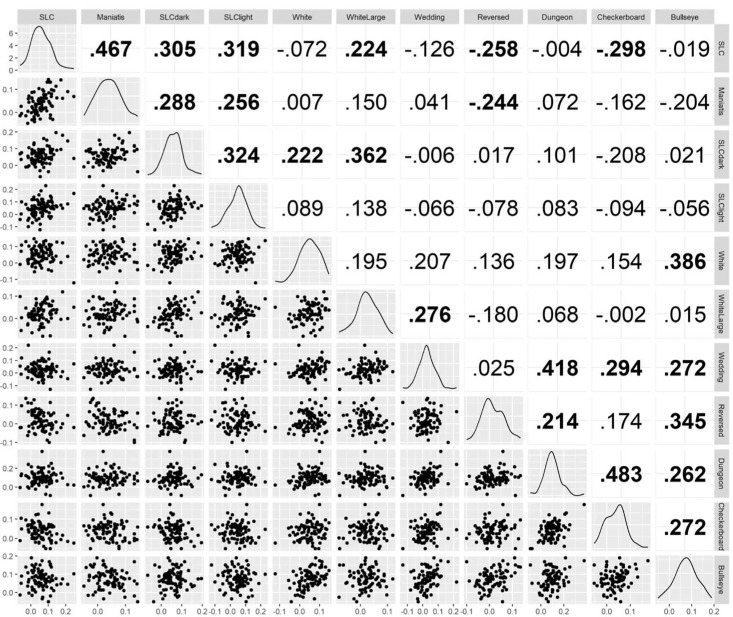
The correlation coefficients (upper triangle) and the scatter plots (lower triangle) of each pair of illusions. The coefficients in boldface indicate significant correlations (*p* < 0.05; no corrections for multiple tests). See the original paper (Nedimović et al., 2022) for details about each illusion.

We analyzed the correlations of the illusion magnitudes. Figure 1 shows correlation coefficients and scatter plots of each pair of illusions. The correlation coefficients ranged from −0.298 to 0.483. Of the 55 comparisons, 22 showed a significant relationship.

We conducted an EFA. First, we determined the number of factors by using very simple structure (VSS) and minimum average partial (MAP) criteria, and a parallel analysis. VSS and MAP suggested a two-factor model and the parallel analysis using the 95th percentile of simulated random data also supported a two-factor model, suggesting that the actual dataset contains two meaningful factors explaining variance beyond what would be expected by chance. We adopted a two-factor model. Then, from the correlation matrix (Figure 1), EFA estimates how much of the variance of each item (i.e., illusion magnitude) is explained by common factors (i.e., commonality). Because the distributions of each illusion's strength were close to normal in this dataset (the diagonal strip in Figure 1), we used maximum likelihood estimation to estimate commonalities. Finally, factor rotation, which simplifies the interpretation of the factors, is used to obtain the factor loading matrix. In the present study, the varimax (orthogonal) rotation was used to determine factor loadings because the factor correlation identified with the oblimin (oblique) rotation was small (*r* = 0.101). Using an orthogonal rotation assumes uncorrelated factors. The independence of the factors was beneficial for evaluating the lightness models, discussed later in the Model Test section.

The factor loading matrix of EFA showed relatively clear separation of two groups of illusions (Table 1). Factor 1 exhibited loadings from illusions with assimilative effects, and Factor 2 exhibited loading that seems to reflect a contrast effect. For instance, the five assimilative illusions showed a high loading in Factor 1 (i.e., Wedding, Reversed, Dungeon, Checkerboard, and Bullseye), and the four SLCs showed that in Factor 2. The two White's illusions (White's and WhiteLarge) demonstrated mixed effects on both factors, with WhiteLarge having more Factor 2 than Factor 1. This is understandable because the targets in WhiteLarge share long edges with the flanking bars, which supposedly exert a contrastive effect on the appearance of the targets (Taya, Ehrenstein, & Cavonius, 1995; Todorović, 1997). Although Nedimović et al. (2022) argued that the illusions in this class should consist of the reverse contrast phenomenon and the assimilation phenomenon, the data-driven classification based on the correlations did not agree with this theoretical classification.

**Table 1.** The factor loading matrix of the data in Nedimović et al. (2022). The colors of the cells representing the factor loadings correspond to their values: orange for positive, and light blue for negative. The rightmost column indicates the commonalities (the sum of the squared factor loadings) of each illusion.

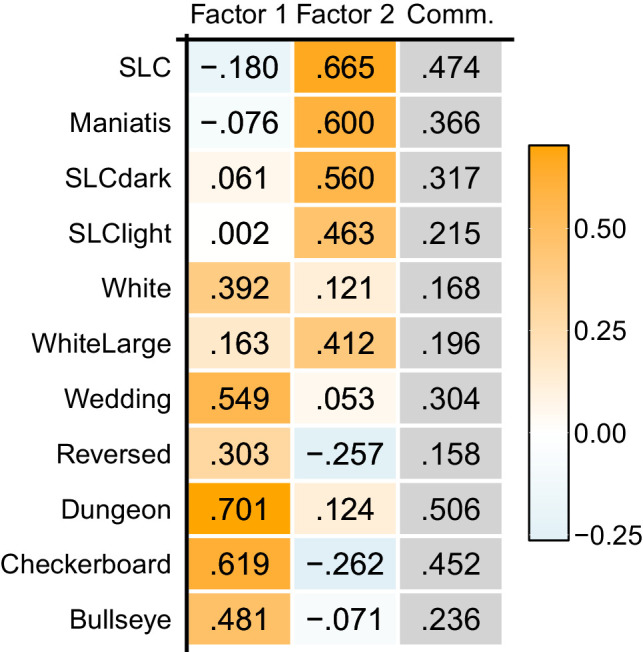

### Discussion

The analysis of a public dataset (Nedimović et al., 2022) demonstrated that the individual differences in the magnitudes of lightness illusions are sufficiently informative for the classification of them. EFA indicated two classes, each composed of phenomenologically similar illusions. Factors 1 and 2 seem to correspond to assimilative and contrastive illusion groups, respectively. This result supports the effectiveness of EFA in classifying illusions. The fact that the four SLC variants consistently showed high loadings to Factor 2 agrees with previous studies that found robust intra-correlations of geometrical illusions (Cretenoud, et al., 2020; Cretenoud et al., 2019; Grzeczkowski, et al., 2017). Although those studies also found that inter-illusion correlations are generally weak (Cretenoud et al., 2019; Grzeczkowski, et al., 2017; Shaqiri et al., 2019), the current analysis, focusing specifically on lightness illusions, detected the similarity among assimilative illusions.

The factor structure indicated by the EFA may need consideration in the evaluation of lightness models. Nedimović et al. (2022) treated 11 illusions as eight items, but the classification by the EFA did not agree with this itemization. In the following two experiments, we examined larger image sets with larger samples and performed model evaluations based on the results (Model Test section).

## Experiment 1

Recognizing that online experiments and EFA can uncover an interpretable factor structure, we conducted our online experiment with a larger dataset on a commonly used set of illusions to examine their relationship. Here, we employed a set of illusions used by Robinson et al. (2007). They sought to extend an existing model, ODOG (Oriented Difference of Gaussian model; Blakeslee & McCourt, 1999), and proposed two new models: LODOG (Locally-normalized ODOG) and FLODOG (Frequency-specific Locally-normalized ODOG). Robinson et al. compared these models using the illusions that had been used to test ODOG (Blakeslee & McCourt, 2004; Howe, 2005; McCourt, 1982) and the number of successful predictions for them. This illusion set was also employed by a subsequent study (Zeman et al., 2015), with a substantial portion comprising variants of White's effect, suggesting redundancy. These model comparisons may cause a bias to White's effect and result in an unfair evaluation of models.

### Methods

**Figure 2.**
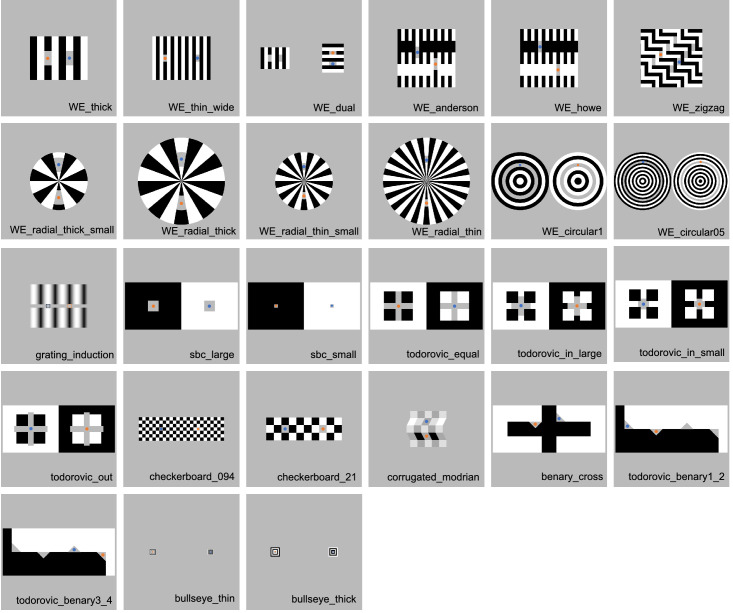
Twenty-seven illusions used in Experiment 1. Blue and orange dots (or frames) indicate target areas. The target areas with blue are those where the average adjustments were darker in this experiment, and those with orange are where the adjustments were lighter. These dots were not shown in the experiment. We excluded WE-circular0.25 and Checkerboard-0.16 from Robinson et al. (2007) because the targets were too small or narrow in these two figures to display in online environments.

We recruited participants via Prolific, an online recruiting service (https://www.prolific.co/), and compensated them with £3 for their participation. The participant pool of Prolific consists of individuals who are 18 years old or older and reside in most OECD countries plus South Africa. We recruited 270 participants, 10 times the number of items under study (Figure 2).

**Figure 3.**
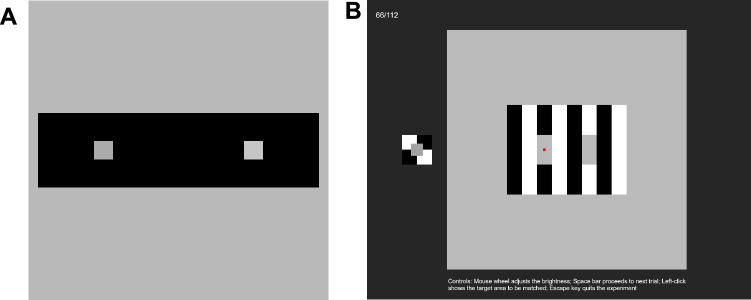
(**A**) The filler image. The right small square was physically lighter than the left. (**B**) An example of the display in the experiment. The count of trials (top left) and an instruction (bottom) were indicated throughout the experiment. The red dot on the figure indicated the target area.

We created stimuli using Stimupy (Schmittwilken et al., 2023). Stimupy is a Python package to design stimuli for vision sciences, and it has a module to precisely recreate stimuli used by Robinson et al. (2007). Although Stimupy provides all illusions used in Robinson et al., we excluded two illusions because the targets were too small or narrow in these two figures to display in online environments. We ultimately used 27 stimuli (Figure 2). Each pixel was represented with a value ranging from zero to one. The targets and the gray surroundings of the illusions were represented with a value of 0.5 in the originally produced images, but because we converted the original images to correct gamma, the gray (0.5) was converted to 0.73 in the experiment. We assumed that the gamma value of monitors was 2.2 (raised to the power of 1/2.2). In most illusions, the other areas were represented with zero (black) and one (white), which remained unaffected by the gamma correction. Corrugated-Mondrian (Adelson, 1993) and Grating-Induction (McCourt, 1982) were the exceptions. We used an additional image (Figure 3A) as a filler stimulus, where two small square patches with different luminance levels were embedded in a black rectangle. The luminance levels of the two patches were approximately 0.67 and 0.78. This luminance difference was considered noticeably large, so the filler image was used to exclude participants who could not detect this difference.

The experiment was created with PsychoPy3 (Peirce et al., 2019) and was hosted on Pavlovia (https://pavlovia.org/), an online server for JavaScript-based programs. The participants accessed the experiment via their PCs; the use of tablets or phones was prohibited. A stimulus was displayed in the center of the screen in a square that occupied 80% of the screen's vertical length (Figure 3B). An adjustable patch on a white-black checker background was presented on the left of the stimulus. The center of the patch was located at a position shifted 50% of the screen's vertical length to the left from the monitor's center. The side lengths of the adjustable patch and the checkered background were 4% and 10% of the screen's vertical length, respectively. Initially, the patch was displayed with a luminance level randomly selected from a uniform distribution ranging from 0.58 to 0.85. The participants could manipulate its luminance with a mouse wheel in a range of 0.39 to 0.94. The amount of patch luminance change in response to the mouse wheel operation was also gamma-corrected to ensure a consistent amount of luminance change on the screen. The task was to adjust the luminance of the patch to that of a target area in the stimulus, indicated by a red dot at the beginning of a trial. The red dot appeared again when the participant left-clicked. When the participants were satisfied with their adjustments, they pressed a space key and started the next trial after a 0.5-second blank during which only dark background was presented. Each stimulus was presented either in the orientation shown in Figure 2 or rotated by 180°. The entire session was composed of 112 trials (28 images × 2 rotations × 2 targets), whose order was randomized. The duration of the entire session was about 20 minutes. Before the session, the participants were instructed to “try to keep a constant distance from the screen throughout the experiment.” To motivate the participants to exert effort, we informed them that compensation might be withheld for “extremely inaccurate or random” task performance, although in reality, compensation was provided in all cases. The experimental program can be accessed via https://run.pavlovia.org/Kobayashi/rhs, and the stimulus set is stored in the supplemental material in Open Science Framework (see Supplement).

### Results

Differences of average adjustments for two targets were defined as the illusion magnitudes (orange minus blue in Figure 2, to ensure that the averaged illusion magnitudes remain consistently positive across all figures). From 270 participants' data, we excluded 28 participants who produced illusion magnitudes falling outside the range of ±3 standard deviations for any of the 27 illusion items. Differences of adjustments for two squares in the filler image were also calculated (lighter minus darker), and 23 participants were excluded because they exhibited negative values. The remaining 219 participants were analyzed. These criteria for exclusion had been determined before conducting the experiment.

Table 2 shows the summary of the magnitudes of observer responses. The magnitudes ranged from 40.85 (WE-Circular05) to 0.078 (WE-Howe). All the illusions showed a substantial and statistically significant illusory effect, except for WE-Howe (Howe, 2005), which we included as a control and will be excluded in the following analyses. The rightmost column indicates intraclass correlations (ICC_2,k_), which examine the reliability of the measurements (Cretenoud et al., 2019). Each ICC was calculated using two magnitudes derived from two rotation conditions in each illusion. Therefore, the two measurements were not exact repetitions but ICCs were generally high. A figure showing inter-illusion correlations, scatter plots, and distributions (equivalent to Figure 1) can be found in the supplemantal material in the Open Science Framework.

**Table 2.** Summary of the results. *Notes:* Mean and SD are based on magnitudes ranging from 0 to 255. ICC indicates ICC_2,k_.

	Mean	*SD*	*t*	*p*	ICC
WE_thick	15.95	18.79	12.56	<0.001	0.63
WE_thin_wide	21.85	20.44	15.81	<0.001	0.68
WE_dual	12.53	16.48	11.26	<0.001	0.49
WE_anderson	14.11	17.53	11.91	<0.001	0.62
WE_howe	0.78	19.06	0.60	0.547	0.66
WE_zigzag	11.81	18.31	9.55	<0.001	0.59
WE_radial_thick_small	7.68	17.57	6.47	<0.001	0.56
WE_radial_thick	11.01	18.25	8.92	<0.001	0.61
WE_radial_thin_small	16.93	19.49	12.85	<0.001	0.51
WE_radial_thin	18.04	21.00	12.71	<0.001	0.72
WE_circular1	32.21	21.73	21.93	<0.001	0.65
WE_circular05	40.85	25.14	24.04	<0.001	0.79
Grating_induction	14.82	22.85	9.60	<0.001	0.68
Sbc_large	8.14	21.34	5.65	<0.001	0.64
Sbc_small	9.40	22.30	6.24	<0.001	0.57
Todorovic_equal	23.38	20.03	17.27	<0.001	0.73
Todorovic_in_large	19.06	18.68	15.10	<0.001	0.66
Todorovic_in_small	13.75	15.49	13.14	<0.001	0.53
Todorovic_out	21.26	20.73	15.18	<0.001	0.68
Checkerboard_094	35.69	24.10	21.91	<0.001	0.73
Checkerboard_21	24.74	17.66	20.73	<0.001	0.67
Corrugated_mondrian	13.32	20.47	9.63	<0.001	0.66
Benary_corss	6.04	16.73	5.35	<0.001	0.45
Todorovic_benary1_2	7.05	18.36	5.68	<0.001	0.64
Todorovic_benary3_4	3.94	16.27	3.58	<0.001	0.52
Bullseye_thin	25.41	21.54	17.46	<0.001	0.72
Bullseye_thick	36.37	25.21	21.46	<0.001	0.73
Filler	—	—	—	—	0.31

The Kaiser-Meyer-Olkin's measure of sampling adequacy was 0.77 for all the data, and the minimum measure in each item was 0.51 in Todorović-Benary3-4. We interpreted this result as indicating that the sample deserved a factor analysis. The VSS and MAP criteria indicated seven and three factors, respectively. A parallel analysis using maximum likelihood estimation suggested three factors. Based on these criteria, we adopted a three-factor model. Table 3 shows the resultant factor loadings with the varimax rotation (the oblimin rotation also showed similar results with factor correlations falling within a range from 0.035 to 0.353). Some illusion pairs phenomenologically similar (e.g., SBC-large and SBC-small, WE-circular0.5 and WE-circular0.25, etc.) showed similar loadings, and this suggests that the EFA could extract reasonable correlations. The three factors accounted for 27.8% of the total variance (i.e., 27.8% of the whole data were explained by the common factors).

**Table 3.** The factor loading matrix obtained in Experiment 1. The cells are colored in the same manner as in Table 1.

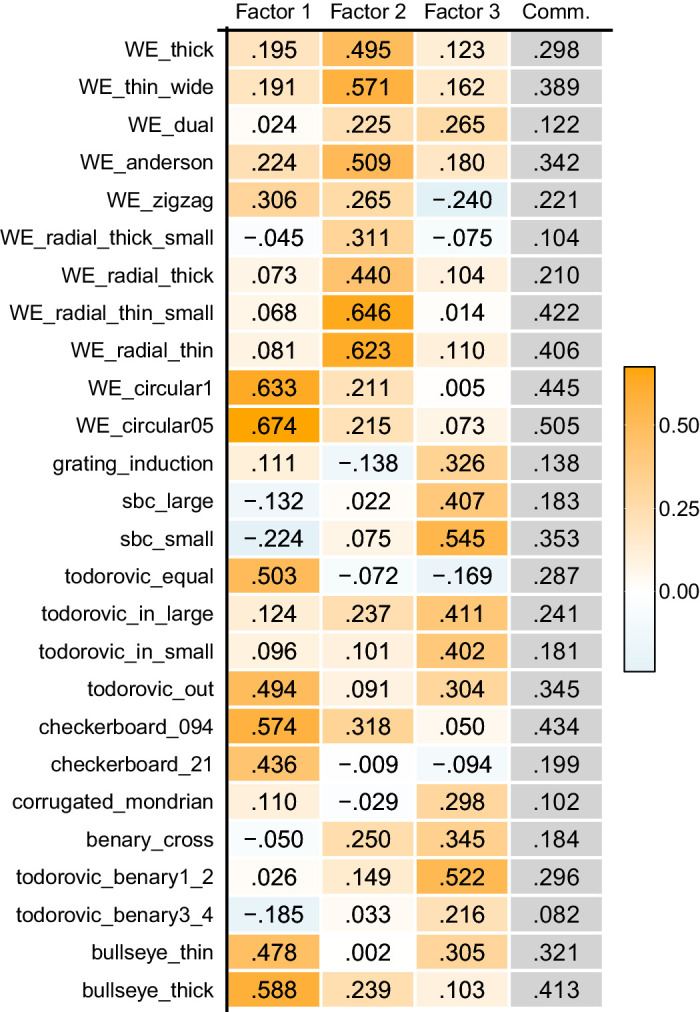

### Discussion

Experiment 1 confirmed that correlations of the illusion magnitudes can identify interpretable common factors from a set of lightness illusions. The Factors seem to correspond to particular aspects of the visual illusions in the presentation set: Factor 1 seems to correspond to assimilation effects; Factor 2, T-junctions/White's effects; and Factor 3, contrast effects. All illusions that showed Factor 1 as their primary loadings (WE_zigzag, WE-circular 1, WE-circular 05, Todorović-equal, Todorović-out, Checkerboard 094, Checkerboard 21, Bullseye-thin, and Bullseye-thick) were all assimilative. The illusions with Factor 2 as the primary loading (WE-thick, WE-thin-wide, WE-Anderson, WE-radial-thick-small, WE-thick, WE-radial-thin-small, and WE-radial-thin) were variants of White's effect (Anderson, Todorović, & Grossberg, 2001; Howe, 2005; Yazdanbakhsh, Arabzadeh, Babadi, & Fazl, 2002). WE-dual and Todorović-in-large, illusions also considered as variants of White's effect (Todorović, 1997), also showed moderate loadings on Factor 2. The illusions with Factor 3 as the major factor showed contrast effects (list). While some illusions to which T-junctions appear relevant also demonstrated moderate to high loadings on Factor 3 (WE-dual, Todorović-in-large, Todorović-in-small, Benary-cross, and Todorovic-Benary 1_2), all four illusions explainable by simple contrast from immediate surroundings primarily loaded on Factor 3 (SBC-large, SBC-small, Grating-induction, and Corrugated-Mondrian). The present experiment with a large sample size revealed that three simply interpretable factors underpin 26 lightness illusions.

The resultant classification offered some insights into lightness perception. First, as in the data of Nedimović et al. (2022), a factor for assimilation and that for contrast were extracted as independent factors. Some people may assume that a participant who perceives strong assimilation effects is likely to perceive weaker contrast effects, but these two illusion groups were not negatively correlated. They are not the other sides of the same phenomenon but are more likely to be independent processes. Second, it was suggested that White's effect has a distinctive characteristic. The substantial number of stimuli related to White's effect in this set likely increased the chances of identifying an independent factor for it, but it was intriguing that the empirical data indicated its uniqueness distinguished from assimilation or contrast. This factor seems to represent a process for T-junctions that modulates contrast effects from neighboring regions (Anderson, 1997; Blakeslee, Padmanabhan, & McCourt, 2016; Taya et al., 1995).

Moreover, the present result also suggested that illusions apparently rooted to the same phenomenon may actually be driven by different processes. For example, while Todorović-in-small, Todorović-in-large, Todorović-equal, and Todorović-out appear a continuum of similar illusions, two of them exhibited high loadings on the factor for contrast, and the other two were dependent more on the factor for assimilation. This is consistent with a T-junction-based explanation proposed by the study that originally introduced these figures (Blakeslee & McCourt, 1999). Another example is circular versions of White's effect (WE-circular 05 and WE-circular 1). They were introduced to demonstrate that an illusion similar to White's effect arises even without T-junctions (Howe, 2005). However, the present experiment suggested that the illusory effect of these figures largely derives from a process different from the original White's effect, and again supported the idea that T-junctions characterize White's effect. The present analysis was particularly insightful in deepening the understanding of the cause behind apparently similar illusions.

It should be noted that the total variance of the three factors was not considerably large (27.8%). Even though the loadings on the three factors were significantly higher than those from random data, some illusions indicated the uniqueness (i.e., a value obtained by subtracting the commonalities from one) exceeding 80%. This partly hints at the independence of each illusion. However, a portion of the uniqueness may be attributed to uncontrolled observation environments (since conducted online) rather than to the inherent attributes of the illusions. Moreover, there was variability in the level of uniqueness, questioning the assumption by the traditional model evaluation (i.e., equally counting all the illusions as one). More importantly, the explained variance depends on a sample size and the number of items. The larger the sample size and the more items used, the more likely that low commonality is obtained (Gordillo, Cretenoud, Garobbio, & Herzog, 2023). A further examination of the illusions’ independence will be discussed in the Model Test section.

## Experiment 2

We conducted another experiment using a different set of illusions. Experiment 1 replicated Nedimović et al (2022) but suggested the presence of a third factor that represents White's effects. Here we expand the range of illusions to include some that were not part of the set used by Robinson et al. (2007). The goal is to explore a process for establishing a standard illusion set for model tests (Murray, 2021; Schmittwilken et al., 2023).

### Methods

Experiment 2 was also conducted online using Prolific and Pavlovia. The sample size was determined to be ten times the number of illusions used. We aimed at 140 participants but obtained 141 people's data due to a technical reason. A total of £2 was compensated for participation. There was no participant overlap between Experiment 1 and Experiment 2.

**Figure 4.**
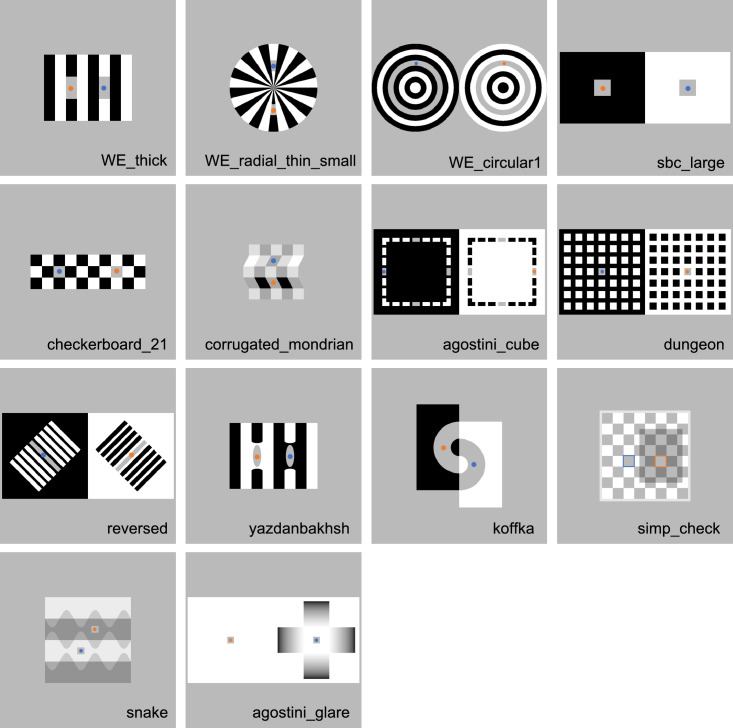
Fourteen illusions used in Experiment 2. Areas marked with orange were perceived as lighter than those with blue in the experiment. The first six illusions were used in Experiment 1 as well. Following are details of the newly included ones. Agostini-cube) An illusion thought to be caused by perceptual grouping between gray targets and aligned white/black rectangles (Agostini & Galmonte, 2002a). Dungeon) An illusion thought to be caused by perceptual grouping between targets and surrounding white/black squares (Bressan, 2001). Reversed) An illusion thought to be caused by perceptual grouping between targets and aligned white/black gratings (Economou et al., 2007; Gilchrist & Annan, 2002). Yazdanbakhsh) An illusion of a similar design to White's effect but without T-junction (Yazdanbakhsh et al., 2002). Koffka) Koffka's ring created by Adelson (2000). Simp-check) A simplified version of Adelson's (1995) checker shadow illusion (Blakeslee & McCourt, 2012). Snake) A contrastive illusion caused by shadow impression (Adelson, 2000). Agostini-glare) An illusion caused by luminance gradient. Immediate surroundings of the two targets are of equal luminance, but the remotely surrounding luminance gradients darken the appearance of the right patch (Agostini & Galmonte, 2002b).

The experimental settings were almost the same as those of Experiment 1 except for the illusions set (Figure 4). First, we included two representative illusions from each of the three classes suggested in Experiment 1: WE-thick and WE-radial-thin-small from the class of White's effect; Checkerboard 21 and WE-circular 1 from the class of assimilation; and SBC-large and Corrugated-Mondrian from the class of contrast. In addition, we used illusions that are deemed reverse contrast phenomena caused by perceptual grouping (Agostini & Galmonte, 2002a; Agostini & Proffitt, 1993; Bressan, 2001; Economou et al., 2015; Gilchrist & Annan, 2002; Gilchrist et al., 1999). Reverse contrast was not identified as a factor independent of assimilation with the data by Nedimović et al. (2022), but we attempted to examine its independence again. For Agostni-cube, a simplified design by Domijan (2015) was used. In addition, an illusion introduced by Yazdanbakhsh et al. (2002) was used as another example of White-effect variants. Similar to the circular version of White's effect (Howe, 2005), this illusion is also known as a counterexample of the T-junction-based explanation of White's effect (Yazdanbakhsh et al., 2002). We also included three illusions that contain explicitly “shadowed” regions: Koffka, Simp-check, and Snake (Adelson, 2000; Blakeslee & McCourt, 2012; Kobayashi & Kitaoka, 2022). They can be understood as variants of simultaneous lightness contrast, but the explicit shadow enhances the illusion in general. Lastly, Agostini-glare was employed as an illusion caused by a luminance gradient (Agostini & Galmonte, 2002b; Kobayashi, Zavagno, & Morikawa, 2021b; Zavagno, Daneyko, & Liu, 2018). For the details about each illusion, see the caption for Figure 4. The stimuli newly adopted in Experiment 2 were created with the same dimensions as in Experiment 1. Most of them were designed by us from scratch, but the Agostini-cube was created with Stimupy. The entire experiment was composed of 60 trials: 15 stimuli (including the filler) × 2 rotations × 2 targets. The duration was approximately 15 minutes.

### Results

Twenty-three participants were excluded with the same criteria as in Experiment 1, so the data from the remaining 118 participants were analyzed. All illusions showed significant effects in *t*-tests in the expected directions (Table 4), so no items were excluded in the following analysis. The Kaiser-Meyer-Olkin's measures of sampling adequacy were relatively high for all the data (0.68), and the minimum measure in each item was 0.32 in Agostini-glare. ICC_2,k_ in Table 4 indicates that the reliability of the measurements was generally high.

**Table 4.** Summary of the results.

	Mean	*SD*	*t*	*p*	ICC
WE_thick	14.52	21.16	7.46	<0.001	0.64
WE_radial_thin_small	17.50	17.10	11.12	<0.001	0.48
WE_circular1	23.82	24.48	10.57	<0.001	0.77
Sbc_large	11.84	21.28	6.05	<0.001	0.64
Checkerboard_21	22.69	19.31	12.77	<0.001	0.69
Corrugated_mondrian	19.94	20.97	10.33	<0.001	0.66
Agostini_cube	5.82	22.44	2.82	0.006	0.73
Dungeon	24.89	22.89	11.81	<0.001	0.69
Reversed	13.94	23.40	6.47	<0.001	0.72
Yazdanbakhsh	15.97	22.30	7.78	<0.001	0.76
Koffka	7.18	22.18	3.52	0.001	0.65
Simp_check	13.86	27.55	5.46	<0.001	0.70
Snake	21.02	25.23	9.05	<0.001	0.76
Agostini_glare	15.71	16.61	10.27	<0.001	0.62
Filler	—	—	—	—	0.36

We conducted EFA on this dataset. VSS criterion, MAP criterion, and a parallel analysis suggested three, one, and two-factor models, respectively. We examined two- and three-factor models (Table 5). As in Experiment 1, maximum likelihood estimation and the varimax rotation were used for the analyses in the following paragraphs. The common factors in the two-factor model accounted for 26% of the total variance, and those in the three-factor model accounted for 34%. The correlations between the factors when used the oblimin rotation were −0.002 in the two-factor model, and within a range from 0.067 to 0.127 in the three-factor model.

**Table 5.** The factor loading matrices of Experiment 2. Left indicates the two-factor model, and right indicates the three-factor model.

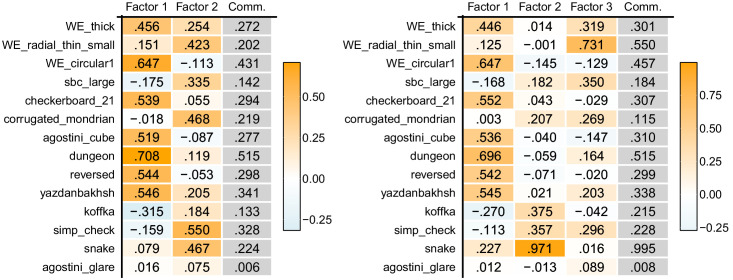

**Figure 5.**
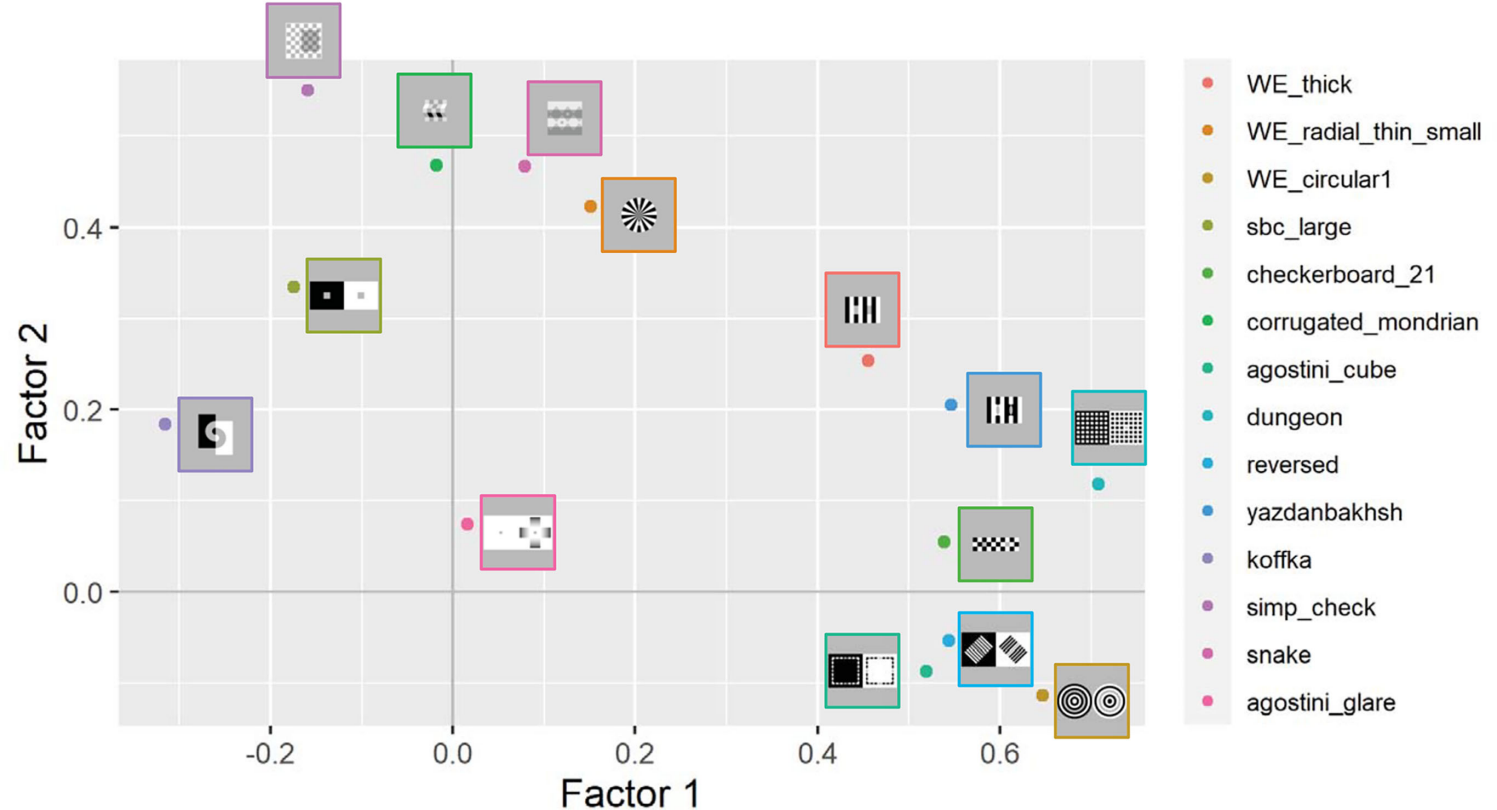
Two-dimensional mapping of the factor loadings in the two-factor model.

In the two-factor model, seven illusions showed relatively high positive loadings to Factor 1 (WE-thick, WE-circular 1, Checkerboard 21, Agostini-cube, Dungeon, Reversed, and Yazdanbakhsh), and five illusions did on Factor 2 (WE-radial-thin-small, SBC-large, Corrugated-Mondrian, Simp-check, and Snake). Koffka and Agostini-glare did not show high positive loadings to either of them. Figure 5 visualizes a two-dimensional mapping of the illusions based on the factor loadings of the two-factor model. In the three-factor model, the Factor 1 was highly loaded by seven illusions (WE-thick, WE-circular 1, Checkerboard 21, Agostini-cube, Dungeon, Reversed, and Yazdanbakhsh), Factor 2 was highly loaded by three illusions (Koffka, Simp-check, and Snake), and Factor 3 was highly loaded by two illusions (WE-radial-thin-small and SBC-large). Corrugated-Mondrian showed weak loadings on Factors 2 and 3, and Agostini-glare did not show high loadings on any of the factors again. The two-factor model seems to indicate factors underlying assimilation and contrast effects, but the three-factor model did not show a structure that separates illusions into assimilation, contrast, and White's effect as in Experiment 1, so a factor for White's effect was not identified in Experiment 2.

### Discussion

We focus on the results of the parallel analysis in this paper and will mainly discuss the two-factor model. The results roughly separated the current illusion set into contrast and assimilation classes, as in the analysis of the data from Nedimović et al. (2022). Although reverse contrast was considered distinct from assimilation (Agostini et al., 2020), illusions that are thought to be largely driven by reverse contrast (Dungeon, Reversed, and Agostini_cube) exhibited similar patterns to those associated with assimilation (e.g., Checkerboard-21). This result implies no clear difference between reverse contrast and assimilation, but the former has been considered to be caused by perceptual grouping with remote regions, while the latter has been associated with lower-level information (e.g., high spatial frequency). It is plausible that both reverse contrast (perceptual grouping) and assimilation (induced by immediate surrounding areas and repetitive patterns) drive illusory effects in the present stimuli (Dungeon, Reversed, and Agostini_cube) in the same direction but the latter is markedly greater (Agostini, Murgia, & Galmonte, 2014; Agostini et al., 2020). This does not deny the role of perceptual grouping in lightness perception. With an appropriate design of an illusion purely driven by grouping (Agostini et al., 2020), we would be able to observe an independent factor (Economou et al., 2015; Kaneko & Gilchrist, 2020).

In Experiment 2, we did not identify an independent factor for White's effect. In the two-factor model, WE-thick exhibited a high loading on Factor 1, which seemingly represents the factor for assimilation, and WE-radial-thin-small was more related to Factor 2, which seemed to reflect the contrast effect. However, it is dependent on the composition of the stimulus set and the magnitude of measurement noise whether an underlying factor can be found. Since the present set included much less White-effect related illusions than in the set in Experiment 1, the variance of data brought by White's effect may not have been large enough to establish an independent factor. Moreover, WE-thick showed a moderate loading (>0.2) on Factor 2, unlike the other assimilative illusions, and both WE-thick and WE-radial-thin-small are plotted in proximity to each other within the first quadrant in Figure 5. This partially supports the notion that White's effect is not simply a contrast or assimilation phenomenon. Unlike Experiment 1, the evidence for the independence of White's effect is inconclusive in Experiment 2, but null results in EFA do not significantly undermine the finding in Experiment 1.

We aimed to examine whether the illusions containing “shadowed” areas show their characteristic tendency. The specific factor for the process of shadow was not extracted in the two-factor model, but Factor 2 in the three-factor model seems to reflect it, showing high loadings from Snake, Koffka, and Simp-check. Considering that SBC-large exhibited its primary loading on Factor 3, Factor 2 should not be considered a factor for a simple contrast effect. It is well known that shadow-like regions enhance illusory effects (Adelson, 2000; Blakeslee & McCourt, 2012), and the present results may have shown this factor.

The observed independence of Agostini-glare was intriguing. This illusion did not show high loadings on any of the factors in both models and was not significantly correlated with any of the other illusions (see Supplement for correlation matrices). Although this illusion has not been extensively explored in model tests, its independence underscores the need to incorporate it—and perhaps similar illusions—in future model evaluations.

## Model tests

In this section, we examine lightness/brightness models using the outcomes of the two experiments. We here compare three models that are based on early processes of brightness perception: ODOG, LODOG, and FLODOG (Blakeslee & McCourt, 1999; Robinson et al., 2007). They have been known as models that succeeded in explaining White's effect without explicitly processing T-junctions and depth information. They have been explored by several studies with many illusions and showed both successes and failures (Betz, Shapley, Wichmann, & Maertens, 2015; Blakeslee & McCourt, 2004; Blakeslee & McCourt, 2012; Economou, Zdravkovic, & Gilchrist, 2007; Kim et al., 2018; Murray, 2020; Nedimović et al., 2022; Zeman et al., 2015). We ran these models on the illusions employed in Experiments 1 and 2, using Multyscale, a Python package including implementations of those models (Vincent & Maertens, 2021a). Robinson et al. (2007) reported these three models’ outputs for their illusion set (i.e., those used in our Experiment 1), but we again ran them on this set because (1) the reported results in Robinson et al. (2007) assumed 32° × 32° image size, which seems implausibly large in the environments employed in the present online experiment, and (2) the target areas indicated in the present experiment were slightly different from those on which the reported results were based. We tested these models on the images assuming their sizes to be 16° × 16°. The pixel values of the input images ranged from 0 to 255.

Table 6 shows the model outputs on the two illusion sets. The outputs for Experiment 1 are similar to those reported in Robinson et al. (2007), but there are slight differences. Using these outputs and the factor loadings obtained in the experiments, we calculated the scores of the models for each factor as follows. First, from a factor loading matrix Λ, Λ_std_ was defined as

$$\Lambda_{std}=\left[\begin{array}{ccc} \frac{\lambda_{11}}{\sum_{k=1}^{m}\lambda_{11}} & \cdots & \frac{\lambda_{1n}}{\sum_{k=1}^{m}\lambda_{kn}}\\ \vdots & \ddots & \vdots \\ \frac{\lambda_{m1}}{\sum_{k=1}^{m}\lambda_{k1}} & \cdots & \frac{\lambda_{mn}}{\sum_{k=1}^{m}\lambda_{k1}} \end{array}\right]$$

where *a_ij_* is the (*i*, *j*) entry of the m*n matrix Λ. Hence, Λ_std_ is the matrix where the sum of each of Λ’s columns is normalized to be one. This normalization equalizes the weights on each factor, which can be unbalanced in a biased illusion set. Then, the factor-based model score was defined as *FBM* *Score* = *R*^⊤^Λ_std_  using the matrix *R*, which binarized version of the model output with zero and one that indicates incorrect and correct predictions of illusion directions, respectively (i.e., the replacement of entries in Table 6 with 1 for red cells and 0 for blue cells). Each value in the resulting FBM score matrix can be interpreted as the percentage of each factor accounted for by the model. Theoretically, the values can exceed one due to negative values in Λ, but they are unlikely in practice. Because the weights on each factor are normalized, FBM scores are not unduly influenced by the composition of the illusion set, even if it primarily includes similar illusions. The scores for the uniqueness of the illusions can also be calculated by simply replacing Λ_std_ with the vector of the uniqueness. Note that the scores for the common factors and those for the uniqueness are in different scales.

**Table 6.** Model outputs for all stimuli used in Experiments 1 (left) and 2 (right). Positive values (correct prediction) are indicated with orange, and negative values (incorrect prediction) are with blue. Values in each table are divided by the output value for WE-thick to be standardized.

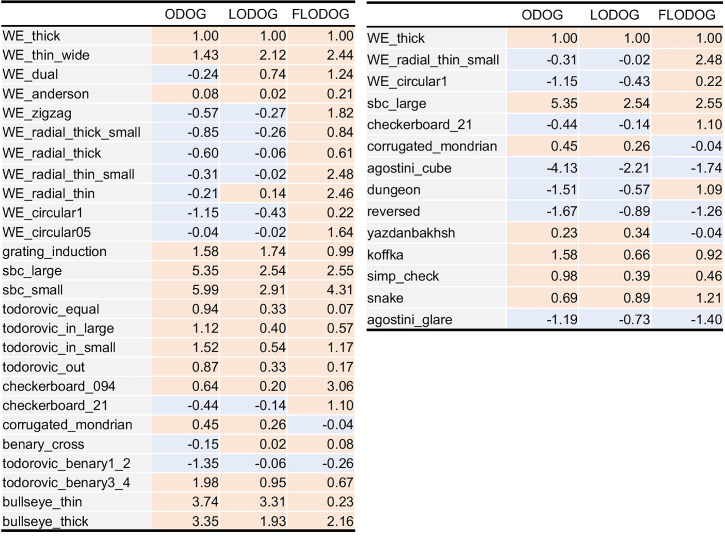

Table 7 shows the models’ scores calculated with the present results. The scores from Experiment 1 indicate that ODOG and LODOG are oriented toward contrast effect. While previous studies reported that these models could explain both contrast and White's effect (Blakeslee & McCourt, 1999; Blakeslee & McCourt, 2004; Blakeslee & McCourt, 2012), the present results suggest that ODOG and LODOG accurately predict White's effect only under specific stimulus designs. The low scores for White's effect, despite their successful predictions for WE_thick (Table 6), suggest limited generalizability, indicating these models could not predict other stimuli containing a White's effect component (e.g., WE_radials). FLODOG scores higher in assimilation and White's effect than ODOG and LODOG; however, its score for contrast is lower, indicating that FLODOG's superiority largely stems from its ability to predict assimilation and White's effect. A similar tendency was also observed in the scores for Experiment 2. ODOG and LODOG's bias toward contrast effects significantly undermines their validity because correct distinction of assimilation and contrast (and other) illusions is one of the crucial abilities that ideal lightness models should possess. The question “To what types of images do humans perceive assimilation, and to what types of images do humans perceive contrast?” is a core question in color/lightness perception studies, but these models fail to provide a satisfactory answer. As for the scores for uniqueness, they were roughly proportional to the simple counts of successfully predicted illusions. Based on these results, the comparative evaluation placing FLODOG over ODOG and LODOG in terms of their performances does not waver even with consideration of correlations, supporting the validity of the simple counts. However, it should be noted that even the best-performing model, FLODOG, exhibits markedly lower scores for assimilation and contrast in Experiment 2 compared to Experiment 1, suggesting potential overfitting to the illusion set used in its initial evaluation (Robinson et al., 2007). EFA enabled the decomposition of the models’ scores into those for underlying factors of illusions, and we revealed that the models based on early visual processes underperformed general expectation.

**Table 7.** Factor-based model scores and counts of correct predictions (Top: Experiment 1, Bottom: Experiment 2).

	Assimilation	White	Contrast	Uniqueness	Count
ODOG	0.59	0.42	0.78	10.89	15
LODOG	0.60	0.61	0.93	13.18	18
FLODOG	0.97	0.98	0.83	17.18	24

ODOG	0.12		0.85	5.34	7
LODOG	0.12		0.85	5.34	7
FLODOG	0.55		0.79	6.46	9

We further examined the quantitative aspect of the model outputs using the present data. Because the output from ODOG-type models is based on an arbitrary scale, it cannot be directly compared to a human response as an absolute measurement (Vincent & Maertens, 2021b). However, a relative comparison is feasible by examining their correlations with various illusions. We conducted Kendall's rank correlation tests for the results of the two experiments respectively. Human responses were defined with the mean values of the illusion magnitudes. For Experiment 1, none of the correlations with the three model's outputs was significant (ODOG: τ*_b_* = 0.089, *p* = 0.541, *n* = 26; LODOG: τ*_b_* = 0.015, *p* = 0.930, *n* = 26; FLODOG: τ*_b_* = 0.089, *p* = 0.541, *n* = 26). No significant result was found in Experiment 2 either (ODOG: τ*_b_* = −0.231, *p* = 0.280, *n* = 14; LODOG: τ*_b_* = −0.165, *p* = 0.451, *n* = 14; FLODOG: τ*_b_* = 0.187, *p* = 0.388, *n* = 14). These tests are not conclusive because they were all post hoc and with small sample sizes (i.e., the number of illusions) and the data points were not independent, but the negative correlations in some pairs provide evidence of the inability of the models to capture human perception. The analysis of correlations suggested that their quantitative predictions cannot yet be considered satisfactory, and with the results described in the above paragraph, the failure by the early-visual-processes-based models suggests the need to incorporate higher-order processing (Kobayashi & Kitaoka, 2022; Murray, 2020).

The framework and data emploused yed in the present study can be applied to test other models. Recent lightness models such as those based on a Markov random field (Kobayashi & Kitaoka, 2022; Murray, 2020) offer a potential solution to the limitations of ODOG related models, by incorporating visual systems’ assumptions about illumination and reflectance. However, they would require modification to accept the larger stimuli used in this study (currently these models are limited to 16 × 16 pixel images). Other recent studies report that convolutional neural network (CNN) models have successfully predicted some lightness illusions (Gomez-Villa, Martín, Vazquez-Corral, Bertalmío, & Malo, 2020; Murray, Brainard, Flachot, & Patel, 2023). Although some simple CNN models correctly predict both assimilation and contrast illusions (Gomez-Villa et al., 2020), more thorough testing with various images is warranted given the limited number of illusions used by Gomez-Villa et al. (2020). The materials (code, data, and stimuli) used for the present tests are all publicly available (see the Supplement section), so any models can be evaluated with the identical pipeline described in this study.

## General discussion

The aim of the present study was to clarify the relationship among various lightness illusions and to enhance the methods used for evaluating computational models. The models have been evaluated with the number of illusions they can successfully predict (Kobayashi & Kitaoka, 2022; Robinson et al., 2007; Zeman et al., 2015), but this naïve method assumes that each illusion is independent. We examined the correlations of the illusion magnitudes to assess the redundancy in sets of illusions and identified several underlying factors. The illusion sets used were not so redundant as to distort the ranking of the three well-accepted models (Blakeslee & McCourt, 1999; Robinson et al., 2007), but the analysis revealed a bias within these models toward contrast illusions, despite their previously reported success in predicting both contrast and White's effect (Blakeslee & McCourt, 1999; Blakeslee & McCourt, 2004; Blakeslee & McCourt, 2012). An additional analysis of the rank correlations between human perceptions and model outputs also demonstrated the models’ inability to quantitatively predict illusion magnitudes. The present study, through a more precise evaluation incorporating the relationships among lightness illusions, identified specific weaknesses in spatial-filtering-based models.

This study made an important contribution not only to model evaluation but also to understanding the mechanisms underlying human lightness perception. Through one analysis of the existing data and two newly conducted experiments, we identified interpretable factor structures. Although the results of EFA should not be regarded as conclusive (Mollon et al., 2017), the present data provide a first empirical foundation for classifying lightness illusions and identifying the major determinants of the perception of lightness. The empirically obtained classification roughly aligned with theoretical classification and well reflected the relationship between the directions of illusions and the contrast polarity of the targets with their immediate surroundings. One important implication from our data is the independence of assimilation and contrast effects; they are not negatively correlated but are instead in an orthogonal relationship. The study also suggested the independence of the process for White's effect from either assimilation or contrast. These identified factors underpin many of the known lightness illusions, and it is suggested that these illusions are essentially different manifestations of the same underlying phenomena, despite their apparent differences. Although averages of illusion magnitudes have traditionally been the primary focus in past research of lightness illusions, the present study highlights the importance of the information contained in covariance. Although this study treated the underlying factors as psychological constructs, future attempts to identify the neural correlates of the underlying factors will significantly enhance understanding of the structure for lightness perception.

The relationship of lightness illusions revealed in the present study offers important suggestions for establishing a standard set of illusions commonly used for model evaluation (Murray, 2021). First, some lightness illusions that have been supposed to reflect the role of perceptual grouping are largely driven by a process for assimilation. For example, Reversed (Figure 4) is one of the illusions considered to be caused by perceptual grouping (Economou et al., 2015; Gilchrist & Annan, 2002), but it showed a high loading on the factor seemingly reflecting assimilation, presumably because the effect of grouping is masked by assimilation induced by the immediate surrounding. To explore the role of grouping in lightness perception, researchers need to create an illusion design purely caused by grouping. Second, White-effect-related illusions need to be included in the set because they suggested difference from contrasts and assimilations. Moreover, the contrast enhancement induced by remote luminance gradients (Agostini & Galmonte, 2002b) is highly independent of other illusions and could not be explained by the three tested models. This fact also compels a greater focus on this illusion and requires it to be used for model evaluation. Overall, the present study suggests that the illusion set should be composed of those driven by contrast, assimilation, White's effect, and contrast enhancement, and necessitates a new design for reverse contrast (illusions caused by perceptual grouping).

Even though the present study used various lightness illusions, there are still many stimuli to be examined in future studies. Correlations with some new illusions that do not seem to be driven by contrast or assimilation are worth examining (Kobayashi & Morikawa, 2019; Kobayashi & Morikawa, 2023). Moreover, because the present study focused on the magnitudes of illusions, we did not used and analyze control figures, which are supposed to cause weaker or no illusory effects despite their resemblance to illusory counterparts (e.g., WE-Howe in Experiment 1 was originally introduced as a weaker version of WE-Anderson). Null predictions for them are also a significant point for models to satisfy. Furthermore, the use of augmented images should also be considered for testing computational models rather than relying on a single image for each illusion. Betz et al. (2015) tested the ODOG model using multiple noise-masked versions of White's illusion. Although the illusion set used in Experiment 1 contained some image pairs that can be considered size-augmented versions of the others (e.g., WE-circular 1 and WE-circular 05), other attributes of images, such as luminance, width-height ratio, or rotation, can also be modified and may have a significant impact on the models’ responses. The models’ robustness to image augmentation is an important evaluation metric. A wider variety of images will further deepen understanding of lightness illusions and models.

The present study provided a new insight into the relationships among general visual illusions. Although correlations among geometrical illusions have been found to be very weak (Cretenoud et al., 2020; Cretenoud, et al., 2020; Cretenoud et al., 2019; Grzeczkowski et al., 2017), our results indicated robust correlations among certain lightness illusions, forming a small number of clusters. Although differences in the perceptual mechanisms underlying lightness and geometric illusions warrant investigation, the criteria used by vision scientists to define distinct illusions should also be considered. Unlike some classic geometrical illusions, where the critical visual components are well defined (e.g., surrounding circles in the Ebbinghaus illusion, arrowheads in the Müller-Lyer illusion), the crucial components for lightness illusions are not necessarily clear (Agostini et al., 2020; e.g., immediate contrast, T-junctions, or spatial frequency, etc.). Therefore it may have been difficult to detect commonalities between very similar lightness illusions, leading to their treatment as distinct phenomena. The present results might highlight the potential problem of the variety of “novel” lightness illusions being reported.

Because the present study used online experiments, some portion of the variance in the datasets may have been introduced by the variety of observation environments. Especially, differences of visual extents should differentiate frequencies of patterns in some illusions, and this difference may have influenced the magnitudes and directions of those illusions. Based on the fact that a higher frequency of a repetitive pattern (per visual degree) enhances assimilation (Blakeslee & McCourt, 2004), the loadings of the assimilation factor might have been moderately inflated due to the potential variety in pattern frequencies introduced by uncontrolled stimulus sizes. For further examination of the relationships of the lightness illusions, controlling visual extents in online environments (Li, Joo, Yeatman, & Reinecke, 2020) can be a key point.

## Conclusion

There are now several computational models to predict human lightness perception; we believe that evaluation of these models is now as important as inventing a new model. In this study, we collected large datasets of the magnitudes of various lightness illusions and used the correlations of the magnitudes to clarify that those illusions can be classified into a few independent groups. The results of classification served as useful information for a more detailed examination of models, focusing on which underlying mechanisms they can (or cannot) account for and to what extent. The present model test revealed that some well-accepted models exhibit a strong bias toward the contrast effect while failing to adequately explain the assimilation effect. This study highlights the need to consider underlying processes rather than focusing solely on individual illusions, and the present findings will contribute to a rigorous theory of human lightness perception.

## Supplementary Material

Supplement 1

## Footnotes

1
Gilchrist (2006) argued that Checkerboard and Bullseye illusions can also be interpreted as outcomes of perceptual grouping.
