# Supplementary material for: Relationships among lightness illusions uncovered by analyses of individual differences
Source: J Vis. 2025 Oct 7;25(12):14. doi: 10.1167/jov.25.12.14 (PMC12517106; doi:10.1167/jov.25.12.14)
Supplement: Supplement 1 [file jovi-25-12-14_s001.docx]

# Supplemental information

## Supplemental analyses: Multidimensional scaling

We further conducted multidimensional scaling (MDS) on our data. MDS is a method to visualize the relationships among objects (e.g., illusions) on a low-dimensional space using pairwise dissimilarity data. Its core idea is similar to that of EFA but MDS does not prioritize interpretability and thus relies on fewer assumptions (e.g., normality and linearity). Therefore, it may identify patterns in the data that might be overlooked by EFA. We created dissimilarity matrices by subtracting correlation matrices from one. While both metric and non-metric MDS were conducted, only the metric MDS results are reported below, because the non-metric MDS solution, based only on ranks of pairwise dissimilarities, proved highly unstable due to its sensitivity to initial values.

We adopted three-dimensional visualization to provide a clear and informative representation the results (Figure 6). Each illusion was colored based on its highest factor loading in the EFA results. The data points represented the same group were positioned in proximity, suggesting consistency between EFA and the metric MDS solutions. The clusters of illusions revealed in the present study seem to be robust to the choices of analysis methods. However, using multiple analysis methods (e.g., EFA, MDS, PCA) is valuable when examining multivariate data, as they can reveal different aspects of the underlying structure of visual phenomena (Kaneko et al., 2021).


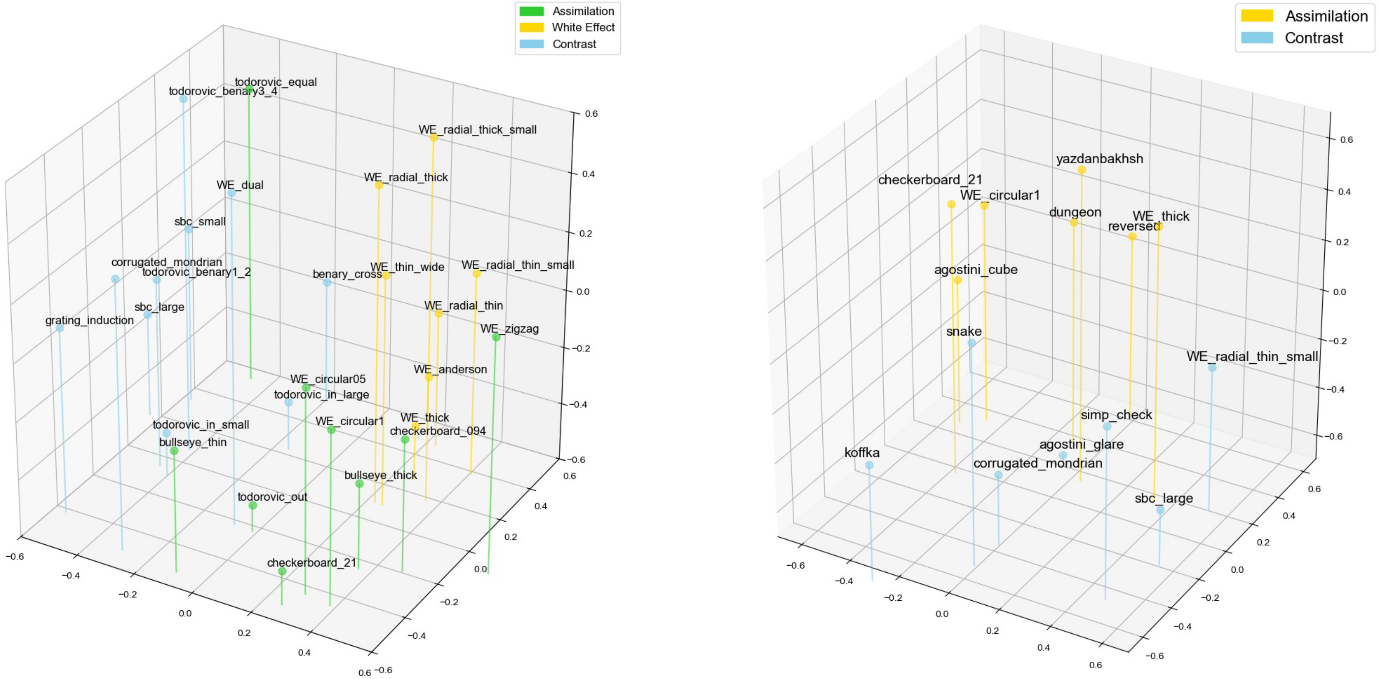


Figure 6. The results of the metric MDS on the data of Experiment 1 (left) and Experiment 2 (right). Readers should note that in MDS, only the relative positions of the points are meaningful, not the rotation or the values of the axes.

## Supplemental materials

Materials used for this study (data, codes, and stimuli) are stored in Open Science Framework: https://osf.io/hq96z/

Experiment programs can be accessed via the links below.

Exp 1: <https://run.pavlovia.org/Kobayashi/rhs>

Exp 2: <https://run.pavlovia.org/Kobayashi/mystims>
